# Congenital hyperinsulinism and panhypopituitarism: a rare combination

**DOI:** 10.1530/EDM-22-0355

**Published:** 2023-05-23

**Authors:** Foram Patel, Ginger Darling, Ahmed Torky

**Affiliations:** 1 Department of Pediatrics, Southern Illinois University, Springfield, Illinois, USA; 2 Department of Pediatrics, Division of Neonatology, Southern Illinois University, Springfield, Illinois, USA; 3 Department of Pediatrics, Division of Pediatric Endocrinology and Diabetes, Southern Illinois University, Springfield, Illinois, USA

**Keywords:** Paediatric, Female, Other, United States, Pancreas, Pituitary, Genetics and mutation, Unique/unexpected symptoms or presentations of a disease, May, 2023

## Abstract

**Summary:**

Neonatal hypoglycemia is a serious condition that can have a major impact on the growing neonatal brain. The differential diagnosis of neonatal hypoglycemia is broad and includes hyperinsulinism as well as panhypopituitarism. The *FOXA2* gene has been involved in the development of the pancreas as well as the pituitary gland. Six cases have been reported thus far with *FOXA2* mutations presenting with variable degrees of hypopituitarism, and only two patients had permanent hyperinsulinism; other cases have been reported with microdeletions in 20p11, the location that encompasses *FOXA2*, and those patients presented with a wider phenotype. A full-term female infant presented with severe hypoglycemia. Critical sampling showed an insulin of 1 mIU/mL, suppressed beta-hydroxybutyric acids, and suppressed free fatty acids. Blood glucose responded to glucagon administration. Growth hormone (GH) stimulation test later showed undetectable GH in all samples, and cortisol failed to respond appropriately to stimulation. Gonadotropins were undetectable at 1 month of life, and MRI showed ectopic posterior pituitary, interrupted stalk, hypoplastic anterior pituitary, cavum septum pellucidum, and diminutive appearance of optic nerves. Whole-exome sequencing revealed a likely pathogenic *de novo c.604 T>C, p.Tyr202His FOXA2* mutation. We expand the known phenotype on *FOXA2* mutations and report a likely pathogenic, novel mutation associated with hyperinsulinism and panhypopituitarism.

**Learning points:**

- *FOXA2* has been shown to play an important role in the neuroectodermal and endodermal development.
- *FOXA2* mutation may lead to the rare combination of hyperinsulinism and panhypopituitarism.
- Patients reported so far all responded well to diazoxide. Dysmorphology may be subtle, and liver functions should be monitored.

## Background

Neonatal hypoglycemia is a serious problem that can lead to developmental disabilities if not addressed properly. Challenges arise from the need to quickly differentiate between the physiologic hypoglycemia related to neonatal transition vs pathologic causes (1).

The pituitary gland plays many vital roles in growth, puberty and reproduction. Additionally, it helps maintain euglycemia through the actions of growth hormone (GH) and steroid. Many transcription factors such as *HESX1, PROP1, POU1F1, LHX3, LHX4, PITX1, PITX2, OTX2, SOX2* and *SOX3* are involved in the development of the gland as well as its differentiation and function (2).

With an estimated incidence of 1 in 50 000 live births (2), congenital hyperinsulinism (CH) is the most common cause of pathologic neonatal hypoglycemia (3). Genetic causes of CH include mutations in the *ABCC8, KCNJ11, HADH* and others that are involved in the pancreatic ẞ cell functions. The combination of hyperinsulinism and panhypopituitarism is quite rare and has only been reported twice in the literature. We report a case of neonatal hypoglycemia due to hypopituitarism and CH found to have a likely pathogenic change in the *FOXA2* that has not been previously reported.

## Case presentation

A female infant was born at an outside hospital at 39 weeks of gestation weighing 3405 g (appropriate for gestational age (AGA)) to a mother with type 1 diabetes. The mother is White and the father is Hispanic. Maternal diabetes was well controlled, and the delivery was uncomplicated. The infant did not require resuscitation in the delivery room. Given the maternal history of diabetes, blood glucose was checked and was <20 mg/dL in point-of-care assessment. Oral correction was attempted; however, i.v. dextrose was initiated due to poor oral feeding. Over the subsequent days, the infant required a GIR of 8–10 mg/kg/min to maintain euglycemia. Multiple attempts to wean IV dextrose failed. The infant was then transferred to our level III neonatal intensive care unit (NICU).

The infant’s physical examination was notable for no anomalies or dysmorphism.

## Investigation

After arrival at our NICU, a critical laboratory sample was sent which showed serum glucose: 35 mg/dL, insulin: 1 μIU/mL, beta-hydroxybutyric acid: 0.1 mmol/L, and cortisol: 0.7 μg/dL. Free fatty acids and GH were undetectable. Glucagon was given after obtaining the sample, and serum glucose increased from 33 mg/dL at baseline to 61 and 70 mg/dL at 20 and 30 min, respectively.

Thyroid functions were checked revealing a thyroid-stimulating hormone (TSH) of 1.94 mIU/L and free T4 0.4 ng/dL (low). Repeat thyroid functions confirmed similar results with TSH: 2.14 mIU/L and free T4: 0.51 ng/dL (low).

A full evaluation of pituitary functions was then performed and included a standard-dose adrenocorticotrophic hormone (ACTH) stimulation test. Cortisol increased from 1.2 μg/dL at baseline to 3.7 and 4.6 μg/dL at 30 and 60 min, respectively.

**Figure 1:**
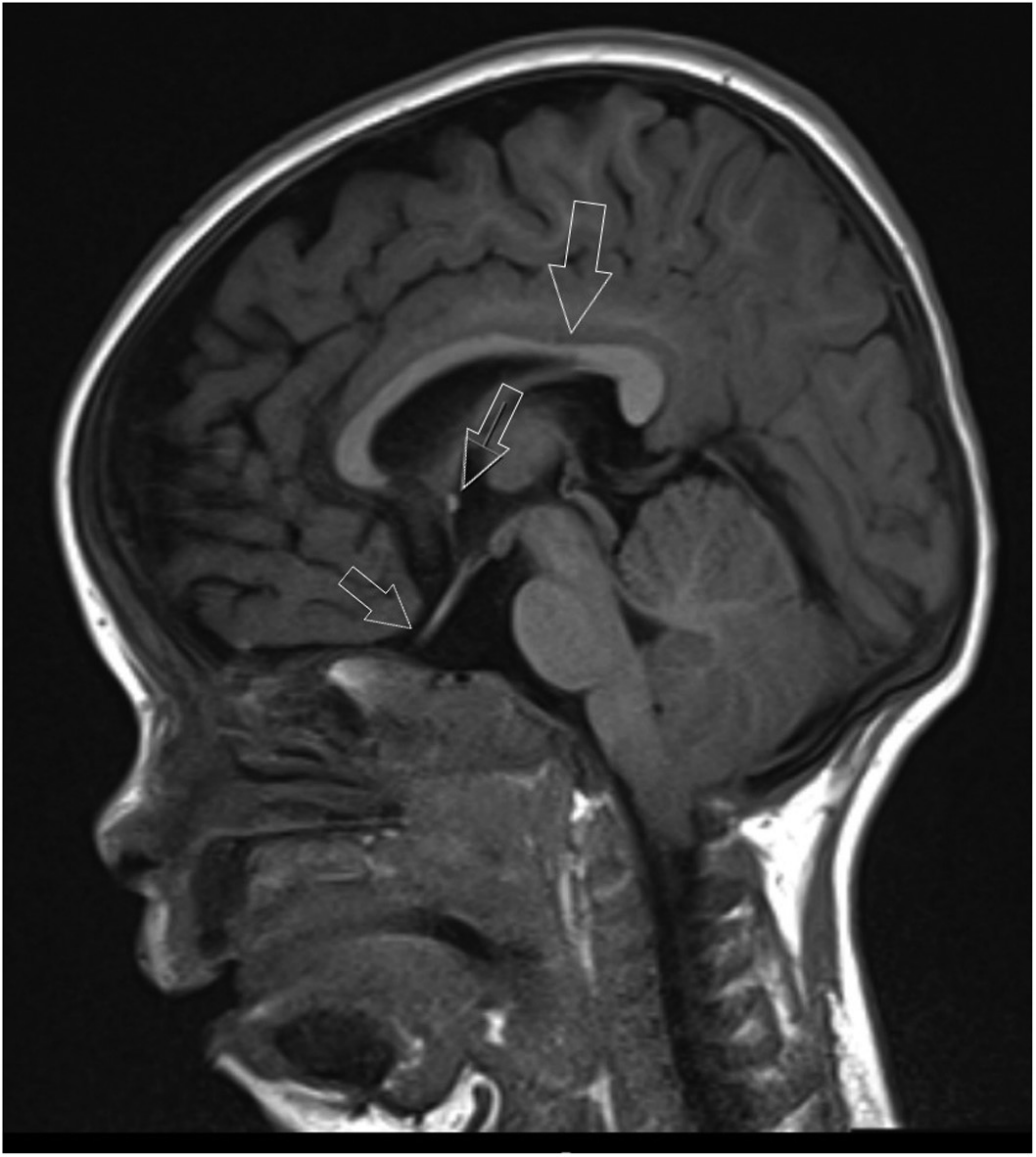
MRI pituitary done at 1 year of age. The arrows from top to bottom point to thin corpus callosum, ectopic posterior pituitary and very short, thin pituitary stalk. The anterior pituitary appears very small in size and the sella appears underdeveloped.

Brain MRI was done (shown in Fig. 1) demonstrating ectopic posterior pituitary, interrupted stalk and hypoplastic anterior pituitary. The rest of the images demonstrated cavum septum pellucidum and diminutive appearance of optic nerves (subsequent ophthalmological evaluation showed normal optic nerves).

Whole-exome sequencing showed a heterozygous novel *de novo* missense mutation in the *FOXA2* gene (c.604 T>C, p.tyr202His). *In silico* analysis predicted the variant to be a pathogenic mutation.

Echocardiogram showed patent foramen ovale (PFO), pulmonic stenosis and tiny patent ductus arteriosus (PDA), all of which subsequently resolved.

At 1 month of life, follicle-stimulating hormone/luteinizing hormone were checked and were undetectable.

At 5 months of age, GH stimulation testing was done by two agents, and GH was undetectable (<0.1 ng/mL) in all samples.

Liver functions were checked on multiple occasions, and aspartate transaminase (AST) was consistently elevated (less than two-fold increase); the highest and most recent level was 55 (13–37), and alanine transaminase (ALT), however, has always been in the normal range. No other gastrointestinal (GI) abnormalities were detected.

## Treatment

After obtaining the critical sampling, diazoxide and diuril were started at 5 mg/kg/day with good response. After ACTH stimulation testing and thyroid function testing, hydrocortisone replacement was initiated, followed by levothyroxine.

As commonly seen in hyperinsulinism patients, this infant demonstrated poor oral feeding skills, and eventually a G-tube was placed prior to discharge.

At 6 months of age, an attempt was made to wean diazoxide; however, hypoglycemia (blood glucose: <55 mg/dL) recurred, and thus she was continued on diazoxide.

## Outcome and follow-up

**Figure 2:**
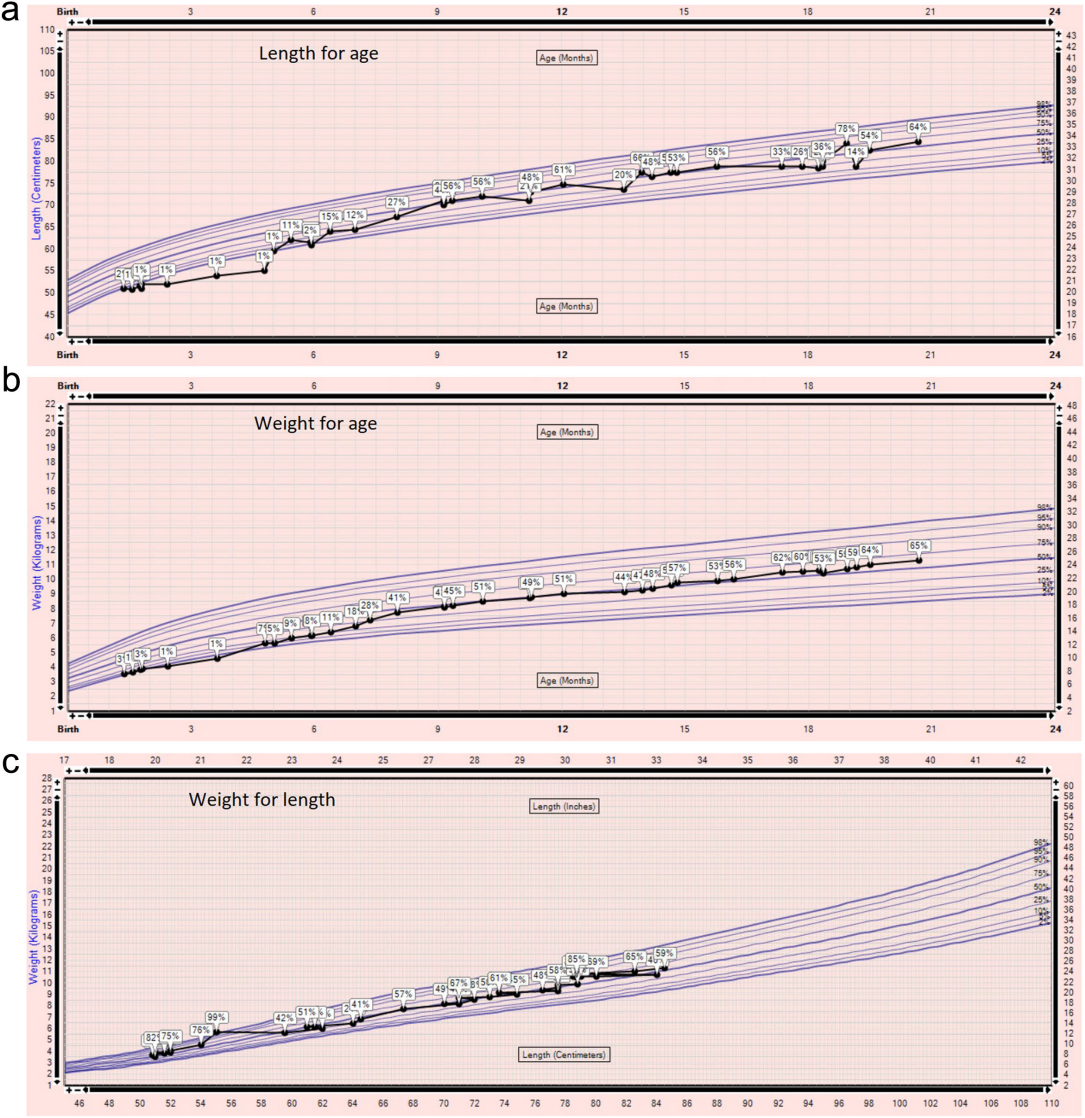
(A) Length for age. (B) Weight for age. (C) Weight for length.

The patient is currently 21 months old and has normal length and weight, as shown in Figs. 2A, B and C. She has developmental delays and is receiving therapy services through early Intervention. Oral aversion has started to improve; however, she remains reliant on G-tube feeding supplementation. She is currently getting PediaSure 1.0 - one can overnight and one can during the day.

She also has been diagnosed with laryngomalacia and obstructive sleep apnea. Repeat MRI at 1 year of age showed Chiari malformation type I with 1 cm cerebellar descent.

She remains on replacement therapy with:

- Hydrocortisone 8.5 mg/m^2^/day,
- Levothyroxine 37.5 μg/day, most recent free T4: 1.07 (0.81–1.35),
- Somatropin 0.25 mg/kg/week, most recent IGF1 is 91 ng/mL (+0.5 SD) and
- Blood glucose remains stable on diazoxide (7.7 mg/kg/day) and diuril.

## Discussion

Hepatocyte nuclear factor-3 family are transcriptional activators. Three of these factors have been identified thus far: *HNF3A*, *HNF3G* and *HNF3B*, also known as *FOXA2*. These transcription factors are crucial for normal liver development (4). In 2015, the first case report was published on a girl who presented at 2 months of age with conjugated jaundice. She was later found to have heterotaxy and biliary atresia. Genetic testing showed a 277 kb heterozygous deletion in 20p11.21 which includes a full gene deletion of *FOXA2.* Her father who was dysmorphic and had situs inversus, polysplenia and panhypopituitarism also had the same deletion. The patient however had polymorphism that is hypothesized to lead to reduced expression of the *NODAL* pathway (another crucial pathway for liver development) (4).

Deletions in 20p11 are rare, and their phenotype varies widely. Despite the variability in deletion sizes reported so far, all patients but one had variable degrees of pituitary affection, with GH deficiency being the most common (4, 6). All patients with pituitary deficiency also showed radiological findings on MRI (5). Hyperinsulinism however was not seen in those patients. Major GI anatomic abnormalities like heterotaxy, hiatal hernia or major organ (spleen/liver/pancreas) affection were seen in seven out of 12 reported cases (5).

Previous studies showed that *FOXA2* plays a role in regulating *PDX1*, a gene vital for pancreatic development. Studies in humans and mice also demonstrate that FOXA2 can regulate very important genes such as *SUR1, HNF4A, HNF1A, HADH* and *GLUT2* (2), and so it is not surprising that *FOXA2* mutations may cause hyperinsulinism. The first report on a patient with panhypopituitarism and hyperinsulinism came in 2017 (2), with a girl presenting at 5 months of age with hypoglycemia and evidence of panhypopituitarism on initial testing. Brain MRI showed similar findings to our patient. Due to persistence of her hypoglycemia, further testing was done showing high insulin and laboratory parameters consistent with hyperinsulinism. She did not tolerate diazoxide due to fluid retention and octreotide caused elevated liver enzymes, and so normoglycemia was maintained by dextrose infusion. The patient showed subtle dysmorphic features. Just as with our patient, she had feeding intolerance and required a gastric tube. GH deficiency was diagnosed at age 1.5 years and at age 3, and she had persistently abnormal liver functions showing ‘chronic inflammation with portal–portal bridging fibrosis’. Similar to our patient, she also had developmental delay. Genetic testing showed *de novo* mutation in *FOXA2* (c.505T>C, p.S169P).

*FOXA2* also plays a vital role in the pituitary development, as evident by animal and human studies showing decreased expression of *SHH, Gli2* and NKX2-2 as a result of *FOXA2* mutations. In 2018, De León *et al.* described a girl of Ashkenazi Jewish descent presenting with neonatal hypoglycemia. Critical sampling showed hyperinsulinism, and testing also showed central hypothyroidism and adrenal insufficiency. GH therapy was started after brain MRI showed findings similar to our patient (3). This patient did show dysmorphic features, and genetic testing showed *de novo* c.770G>T, p.R257L *FOXA2* mutation. It was not reported that the patient had any feeding intolerance or evidence of liver affection.

The six patients reported with *FOXA2* mutations come from different races, and their hypoglycemia was picked up in the first year of life (5). Their degree of hypopituitarism appears to be more severe than patients with 20p11 deletions; all but one patient (8) had three hormonal deficiencies.

Two patients only presented with hyperinsulinism severe enough to warrant therapy (2, 3), two patients had biochemical evidence of hyperinsulinism but they were not treated as per the publications (5, 6) and two patients did not have any biochemical evidence of hyperinsulinism (7, 8). Patients with permanent hyperinsulinism responded to diazoxide, although one did not tolerate it.

Some GI abnormalities were detected but were much less severe than patients with 20p11 deletions, and the most common abnormality was anal stenosis/atresia seen in three patients (5).

Our study reports a likely pathogenic novel mutation in the *FOXA2* gene in a patient presenting with hyperinsulinism and panhypopituitarism. This rare combination has only been seen in patients with *FOXA2* mutations. All patients reported thus far responded to diazoxide. Dysmorphism may be absent or subtle, and GI abnormalities should be investigated.

*FOXA2* has been shown to play an important role in the neuroectodermal and endodermal development. Adding *FOXA2* to genetic testing of neonatal hypoglycemia may be beneficial.

## Declaration of interest

There is no conflict of interest that could be perceived as prejudicing the impartiality of the research reported.

## Funding

This work did not receive any specific grant from any funding agency in the public, commercial or not-for-profit sector.

## Patient consent

Written informed consent for publication of the clinical details and clinical images was obtained from the parent of the patient.

## Patient’s perspective

The weeks after our daughter’s birth were the worst and most confusing weeks of our lives. During that first week before the transfer to a higher level of care, we were told our daughter was slow to transition from womb to life in the outside world. She was not eating and not maintaining her body temperature. She was jaundiced, hypoglycemic, never awake and never cried. I remember holding her, feeling like she wasn’t even alive, wondering if she was going to remain lifeless, and wondering was this my fault because I am type 1 diabetic. When no progress was made in that first week, we asked for a transfer to a higher level of care. Advocating for our daughter is what saved her life. We received her diagnoses, she began treatment, and she discharged after placement of a feeding tube.

Her first year was a struggle, several low blood sugar levels, countless medication dosage adjustments, and no progress with eating or drinking by mouth despite weekly feeding therapy. Also, she was not growing, gaining weight, or meeting any developmental milestones. She was vomiting daily, two to ten times a day with no apparent cause found in GI testing and no medication had been effective in stopping her vomiting. At about six months of age, she started Norditropin and that was the start of improvement for her. She started rolling over, sitting up, became more interactive. Growth hormone stabilized her blood sugars, and she began to grow and gain weight. This medication was life changing for my daughter and we truly believe it made her feel better.

She did continue to projectile and forcefully vomit most of what she ate by mouth or through her gastric tube. Most of her physicians were not concerned because she was gaining weight and her blood sugars were stable. After advocating and pushing for more testing, she was diagnosed with severe gastroparesis. We had already tried medications to speed her GI motility that were not effective, and the other medications had terrible side effects. We decided to try smaller, more frequent tube feedings every two hours. Day one of the new feeding schedule, her vomiting ceased and her eating by mouth increased.

Now, at 2 years of age, our daughter is the healthiest she has been. She is eating many different foods and drinking fluids by mouth. She is beginning to walk independently. She is not talking but communicating with gestures and noises. She is still reliant on her gastric tube since she does not take in enough calories. While we still must remember medication times, tube feedings, watch for signs of struggling and crisis, as well as few other items, these tasks are routine for us. Our daughter is happy and healthy, and we could not be more excited for her future.

## Author contribution statement

AT and GD provided clinical care and followed-up the patient. AT and FP wrote the manuscript. GD reviewed and edited the manuscript. The authors wish to express their gratitude to the family.

## References

[1] Thornton PS Stanley CA De Leon DD Harris D Haymond MW Hussain K Levitsky LL Murad MH Rozance PJ Simmons RA, Recommendations from the pediatric Endocrine Society for evaluation and management of persistent hypoglycemia in neonates, infants, and children. Journal of Pediatrics 2015 167 238–245. (10.1016/j.jpeds.2015.03.057) 25957977 PMC11891912

[2] Giri D Vignola ML Gualtieri A Scagliotti V McNamara P Peak M Didi M Gaston-Massuet C & Senniappan S. Novel FOXA2 mutation causes Hyperinsulinism, Hypopituitarism with Craniofacial and Endoderm-derived organ abnormalities. Human Molecular Genetics 2017 26 4315–4326. (10.1093/hmg/ddx318) 28973288

[3] Vajravelu ME Chai J Krock B Baker S Langdon D Alter C & De León DD. Congenital hyperinsulinism and hypopituitarism attributable to a mutation in FOXA2. Journal of Clinical Endocrinology and Metabolism 2018 103 1042–1047. (10.1210/jc.2017-02157) 29329447 PMC6276717

[4] Tsai EA Grochowski CM Falsey AM Rajagopalan R Wendel D Devoto M Krantz ID Loomes KM & Spinner NB. Heterozygous deletion of FOXA2 segregates with disease in a family with heterotaxy, panhypopituitarism, and biliary atresia. Human Mutation 2015 36 631–637. (10.1002/humu.22786) 25765999 PMC4477513

[5] Kaygusuz SB Arslan Ates E Vignola ML Volkan B Geckinli BB Turan S Bereket A Gaston-Massuet C & Guran T. Dysgenesis and dysfunction of the pancreas and pituitary due to FOXA2 gene defects. Journal of Clinical Endocrinology and Metabolism 2021 106 e4142–e4154. (10.1210/clinem/dgab352) 33999151

[6] Dines JN Liu YJ Neufeld-Kaiser W Sawyer T Ishak GE Tully HM Racobaldo M Sanchez-Valle A Disteche CM Juusola J, Expanding phenotype with severe midline brain anomalies and missense variant supports a causal role for FOXA2 in 20p11.2 deletion syndrome. American Journal of Medical Genetics. Part A 2019 179 1783–1790. (10.1002/ajmg.a.61281) 31294511

[7] Boda H Miyata M Inagaki H Shinkai Y Kato T Yoshikawa T & Kurahashi H. FOXA2 gene mutation in a patient with congenital complex pituitary hormone deficiency. European Journal of Medical Genetics 2019 62 103570. (10.1016/j.ejmg.2018.11.004) 30414530

[8] Stekelenburg C Gerster K Blouin JL Lang-Muritano M Guipponi M Santoni F & Schwitzgebel VM. Exome sequencing identifies a de novo FOXA2 variant in a patient with syndromic diabetes. Pediatric Diabetes 2019 20 366–369. (10.1111/pedi.12814) 30684292

